# Smoothing of, and Parameter Estimation from, Noisy Biophysical Recordings

**DOI:** 10.1371/journal.pcbi.1000379

**Published:** 2009-05-08

**Authors:** Quentin J. M. Huys, Liam Paninski

**Affiliations:** 1 Gatsby Computational Neuroscience Unit, University College London, London, United Kingdom; 2 Center for Theoretical Neuroscience, Columbia University, New York, New York, United States of America; 3 Statistics Department, Columbia University, New York, New York, United States of America; University College London, United Kingdom

## Abstract

Biophysically detailed models of single cells are difficult to fit to real data. Recent advances in imaging techniques allow simultaneous access to various intracellular variables, and these data can be used to significantly facilitate the modelling task. These data, however, are noisy, and current approaches to building biophysically detailed models are not designed to deal with this. We extend previous techniques to take the noisy nature of the measurements into account. Sequential Monte Carlo (“particle filtering”) methods, in combination with a detailed biophysical description of a cell, are used for principled, model-based smoothing of noisy recording data. We also provide an alternative formulation of smoothing where the neural nonlinearities are estimated in a non-parametric manner. Biophysically important parameters of detailed models (such as channel densities, intercompartmental conductances, input resistances, and observation noise) are inferred automatically from noisy data via expectation-maximisation. Overall, we find that model-based smoothing is a powerful, robust technique for smoothing of noisy biophysical data and for inference of biophysical parameters in the face of recording noise.

## Author Summary

Cellular imaging techniques are maturing at a great pace, but are still plagued by high levels of noise. Here, we present two methods for smoothing individual, noisy traces. The first method fits a full, biophysically accurate description of the cell under study to the noisy data. This allows both smoothing of the data and inference of biophysically relevant parameters such as the density of (active) channels, input resistance, intercompartmental conductances, and noise levels; it does, however, depend on knowledge of active channel kinetics. The second method achieves smoothing of noisy traces by fitting arbitrary kinetics in a non-parametric manner. Both techniques can additionally be used to infer unobserved variables, for instance voltage from calcium concentration. This paper gives a detailed account of the methods and should allow for straightforward modification and inclusion of additional measurements.

## Introduction

Recent advances in imaging techniques allow measurements of time-varying biophysical quantities of interest at high spatial and temporal resolution. For example, voltage-sensitive dye imaging allows the observation of the backpropagation of individual action potentials up the dendritic tree [1]–[6]. Calcium imaging techniques similarly allow imaging of synaptic events in individual synapses. Such data are very well-suited to constrain biophysically detailed models of single cells. Both the dimensionality of the parameter space and the noisy and (temporally and spatially) undersampled nature of the observed data renders the use of statistical techniques desirable. Here, we here use sequential Monte Carlo methods (“particle filtering”) [7],[8]—a standard machine-learning approach to hidden dynamical systems estimation—to automatically smooth the noisy data. In a first step, we will do this while inferring biophysically detailed models; in a second step, by inferring non-parametric models of the cellular nonlinearities.

Given the laborious nature of building biophysically detailed cellular models by hand [9]–[11], there has long been a strong emphasis on robust automatic methods [12]–[19]. Large-scale efforts (e.g. http://microcircuit.epfl.ch) have added to the need for such methods and yielded exciting advances. The Neurofitter [20] package, for example, provides tight integration with a number of standard simulation tools; implements a large number of search methods; and uses a combination of a wide variety of cost functions to measure the quality of a model's fit to the data. These are, however, highly complex approaches that, while extremely flexible, arguably make optimal use neither of the richness of the structure present in the statistical problem nor of the richness of new data emerging from imaging techniques. In the past, it has been shown by us and others [18], [21]–[23] that knowledge of the true transmembrane voltage decouples a number of fundamental parameters, allowing simultaneous estimation of the spatial distribution of multiple kinetically differing conductances; of intercompartmental conductances; and of time-varying synaptic input. Importantly, this inference problem has the form of a constrained linear regression with a single, global optimum for all these parameters given the data.

None of these approaches, however, at present take the various noise sources (channel noise, unobserved variables etc.) in recording situations explicitly into account. Here, we extend the findings from [23], applying standard inference procedures to well-founded statistical descriptions of the recording situations in the hope that this more specifically tailored approach will provide computationally cheaper, more flexible, robust solutions, and that a probabilistic approach will allow noise to be addressed in a principled manner.

Specifically, we approach the issue of noisy observations and interpolation of undersampled data first in a model-based, and then in a model-free setting. We start by exploring how an accurate description of a cell can be used for optimal de-noising and to infer unobserved variables, such as Ca^2+^ concentration from voltage. We then proceed to show how an accurate model of a cell can be inferred from the noisy signals in the first place; this relies on using model-based smoothing as the first step of a standard, two-step, iterative machine learning algorithm known as Expectation-Maximisation [24],[25]. The “Maximisation” step here turns out to be a weighted version of our previous regression-based inference method, which assumed exact knowledge of the biophysical signals.

### Overview

The aim of this paper is to fit biophysically detailed models to noisy electrophysiological or imaging data. We first give an overview of the kinds of models we consider; which parameters in those models we seek to infer; how this inference is affected by the noise inherent in the measurements; and how standard machine learning techniques can be applied to this inference problem. The overview will be couched in terms of voltage measurements, but we later also consider measurements of calcium concentrations.

#### Compartmental models

Compartmental models are spatially discrete approximations to the cable equation [13],[26],[27] and allow the temporal evolution of a compartment's voltage to be written as
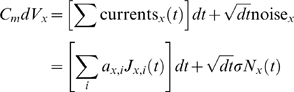
(1)where 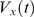 is the voltage in compartment 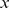, 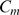 is the specific membrane capacitance, and 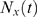 is current evolution noise (here assumed to be white and Gaussian). Note the important factor 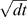 which ensures that the noise variance grows linearly with time 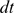. The currents 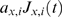 we will consider here are of three types:

- Axial currents along dendrites
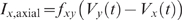
(2)
- Transmembrane currents from active (voltage-dependent), passive, or other (e.g. Ca^2+^ -dependent) membrane conductances
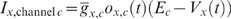
(3)
- Experimentally injected currents
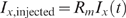
(4)where 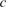 indicates one particular current type (“channel”), 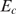 its reversal potential and 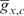 its maximal conductance in compartment 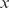, 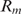 is the membrane resistivity and 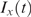 is the current experimentally injected into that compartment. The variable 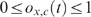 represents the time-varying open fraction of the conductance, and is typically given by complex, highly nonlinear functions of time and voltage. For example, for the Hodgkin and Huxley (HH) K^+^ -channel, the kinetics are given by 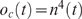, with
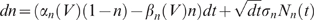
(5)and 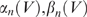 themselves nonlinear functions of the voltage [28] and we again have an additive noise term. In practice, the gate noise is either drawn from a truncated Gaussian, or one can work with the transformed variable 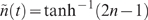. Similar equations can be formulated for other variables such as the intracellular free Ca^2+^ concentration [27].

#### Noiseless observations

A detailed discussion of the case when the voltage is observed approximately noiselessly (such as with a patch-clamp electrode) is presented in [23] (see also [18],[21],[22]). We here give a short review over the material on which the present work will build. Let us henceforth assume that all the kinetics (such as 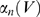) of all conductances are known. Once the voltage is known, the kinetic equations can be evaluated to yield the open fraction 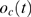 of each conductance 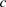 of interest. We further assume knowledge of the reversal potentials 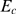, although this can be relaxed, and of the membrane specific capacitance 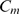 (which is henceforth neglected for notational clarity and fixed at 1 nF/cm^2^; see [29] for a discussion of this assumption).

Knowledge of channel kinetics and voltage in each of the cell's compartments allows inference of the linear parameters 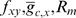 and of the noise terms by constrained linear regression [23]. As an example, consider a single-compartment cell containing one active (Hodgkin-Huxley K^+^) and one leak conductance and assume the voltage 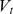 has been recorded at sampling intervals 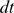 for a time period of 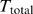. Let 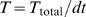 be the number of data points and 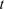 index them successively 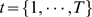:
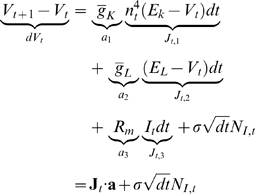
(6)where we see that only 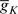, 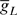 and 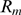 are now unknown; that they mediate the linear relationship between 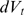 and 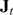; and that these parameters can be concatenated into a vector 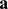 as illustrated in equation 6. The maximum likelihood (ML) estimate of 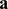 (in vectorized form) and of 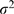 are given by
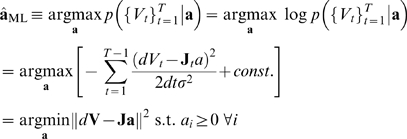
(7)

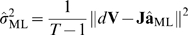
(8)where 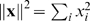. Note that the last equality in equation 7 expresses the solution of the model fitting problem as a quadratic minimization with linear constraints on the parameters and is straightforwardly performed with standard packages such as quadprog.m in Matlab. The quadratic log-likelihood in equation 7 and therefore the linear form of the regression depends on the assumption that the evolution noise 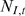 of the observed variable in equation 6 is Gaussian white noise. Parameters that can be simultaneously inferred in this manner from the true voltage trace are 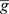, 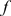, 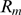, time-varying synaptic input strengths and the evolution noise variances [23].

In the following, we will write all the dynamical equations as simultaneous equations
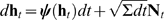
(9)where 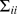 is the evolution noise variance of the 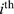 dynamic variable, 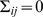 if 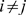 and 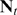 denotes a vector of independent, identically distributed (iid) random variables. These are Gaussian for unconstrained variables such as the voltage, and drawn from truncated Gaussians for constrained variables such as the gates. For the voltage we have 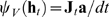 and we remind ourselves that 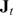 is a function of 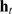 (equation 6).

#### Observation noise

**Figure 1:**
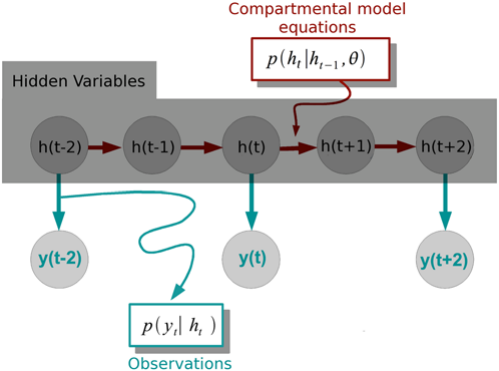
Hidden dynamical system. The dynamical system comprises the *hidden* variables 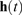 and evolves as a Markov chain according to the compartmental model and kinetic equations. The dynamical system is hidden, because only noisy measurements of the true voltage are observed. To perform inference, one has to take the observation process 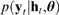 into account. Inference is now possible because the total likelihood of both observed and unobserved quantities given the parameters can be expressed in terms of these two probabilistic relations.

Most recording techniques yield estimates of the underlying variable of interest that are much more noisy than the essentially noise-free estimates patch-clamping can provide. Imaging techniques, for example, do not provide access to the true voltage which is necessary for the inference in equation 7. Figure 1 describes the hidden dynamical system setting that applies to this situation. Crucially, measurements 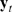 are instantaneously related to the underlying voltage 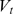 by a probabilistic relationship (the turquoise arrows in Figure 1) which is dependent on the recording configuration. Together, the model of the observations, combined with the (Markovian) model of the dynamics given by the compartmental model define the following hidden dynamical system:
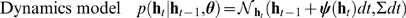
(10)

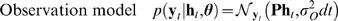
(11)where 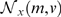 denotes a Gaussian or truncated Gaussian distribution over 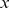 with mean 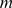 and variance 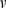 and 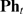 denotes the linear measurement process (in the following simply a linear projection such that 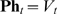 or 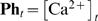). We assume Gaussian noise both for the observations and the voltage; and truncated Gaussian noise for the gates. The Gaussian assumption on the evolution noise for the observed variable allows us to use a simple regression (equation 7) in the inference of the channel densities. Note that although the noise processes are i.i.d., the fact that noise is injected into all gates means that the effective noise in the observations can show strong serial correlations.

**Figure 2:**
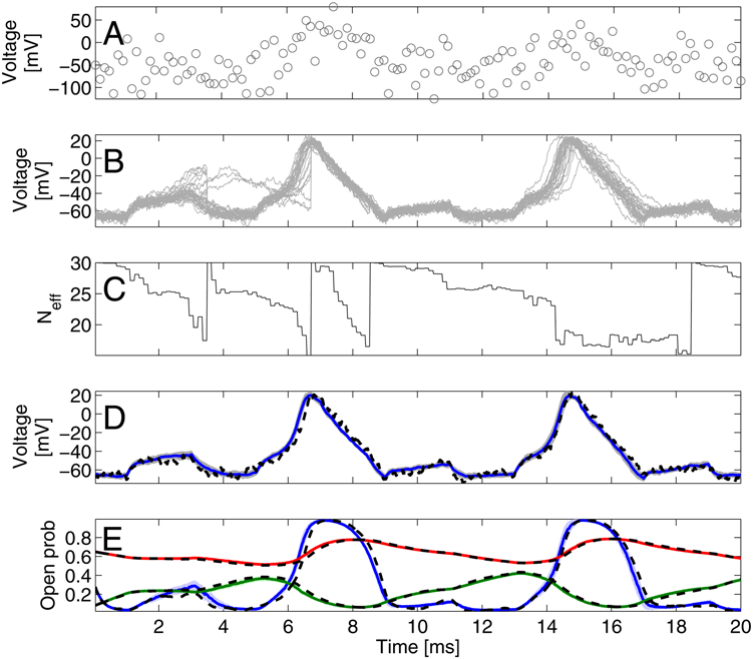
Model-based smoothing. A: Data; generated by adding Gaussian noise (*σ_O_* = 30 mV) to the voltage trace and subsampling every seven timesteps (Δ = 0.02 ms and Δ*_s_* = 0.14 ms). The voltage trace was generated by running the equation 1 for the single compartment with the correct parameters once and adding noise of variance 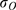. B: Voltage paths corresponding to the 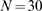 particles which were run with the correct, known parameters. C: Effective particle number 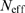. As soon as enough particles have ‘drifted’ away from the data (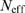 reaches the threshold 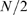), a resampling step eliminates the stray particles (they are reset to a particle with larger weight) all weights are reset to 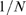 and the effective number returns to 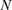. D: expected voltage trace 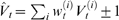 st. dev. in shaded colours. The mean reproduces the underlying voltage trace with high accuracy. E: Conditional expectations for the gates of the particles (mean ±1 st. dev.); blue: HH 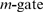; green: HH 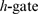; red: HH 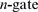. Thus, using model-based smoothing, a highly accurate estimate of the underlying voltage and the gates can be recovered from very noisy, undersampled data.

Importantly, we do not assume that 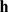 bas the same dimensionality as 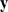; in a typical cellular setting, there are several unobserved variables per compartment, only one or a few of them being measured. For Figure 2, which illustrates the particle filter for a single-compartment model with leak, Na^+^ and K^+^ Hodgkin-Huxley conductances, only 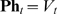 is measured, although the hidden variable 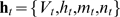 is 4-dimensional and includes the three gates for the Na^+^ and K^+^ channels in the classical Hodgkin-Huxley model. It is, however, possible to have 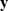 of dimensionality equal to (or even greater than) 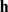. For example, [5] simultaneously image voltage- and [Ca^2+^]-sensitive dyes.

### Expectation-Maximisation

Expectation-Maximisation (EM) is one standard machine-learning technique that allows estimation of parameters in precisely the circumstances just outlined, i.e. where inference depends on unobserved variables and certain expectations can be evaluated. The EM algorithm achieves a local maximisation of the data likelihood by iterating over two steps. For the case where voltage is recorded, it consists of:

1. **Expectation step** (E-Step): The parameters are fixed at their current estimate 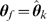; based on this (initally inaccurate) parameter setting, the conditional distribution of the hidden variables 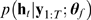 (where 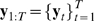 are all the observations) is inferred. This effectively amounts to model-based smoothing of the noisy data and will be discussed in the first part of the paper.
2. **Maximisation step** (M-Step): Based on the model-based estimate of the hidden variables 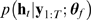, a new estimate of the parameters 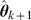 is inferred, such that it maximises the expected joint log likelihood of the observations and the inferred distribution over the unobserved variables. This procedure is a generalisation of parameter inference in the case mentioned in equation 7, where the voltage was observed noiselessly.

The EM algorithm can be shown to increase the likelihood of the parameters at each iteration [24],[25],[30],[31], and will typically converge to a local maximum. Although in combination with the Monte-Carlo estimation these guarantees no longer hold, in practice, we have never encountered nonglobal optima.

## Methods

### Model-based smoothing

We first assume that the true parameters 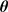 are known, and in the E-step infer the conditional marginal distributions 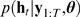 for all times 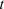. The conditional mean 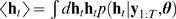 is a model-based, smoothed estimate of the true underlying signal 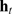 at each point in time 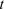 which is optimal under mean squared error. The E-step is implemented using standard sequential Monte Carlo techniques [7]. Here we present the detailed equations as applied to noisy recordings of cellular dynamic variables such as the transmembrane voltage or intracellular calcium concentration.

The smoothed distribution 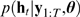 is computed via a backward recursion which relies on the filtering distribution 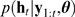, which in turn is inferred by writing the following recursion (suppressing the dependence on 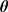 for clarity):
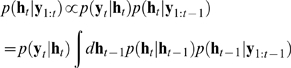
(12)This recursion relies on the fact that the hidden variables are Markovian
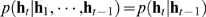
(13)Based on this, the smoothed distribution, which gives estimates of the hidden variables that incorporate all, not just the past, observations, can then be inferred by starting with 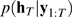 and iterating backwards:
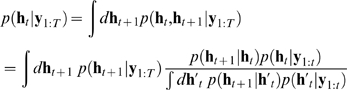
(14)where all quantities inside the integral are now known.

### Sequential Monte Carlo

The filtering and smoothing equations demand integrals over the hidden variables. In the present case, these integrals are not analytically tractable, because of the complex nonlinearities in the kinetics 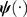. They can, however, be approximated using Sequential Monte Carlo methods. Such methods (also known as “particle filters”) are a special version of importance sampling, in which distributions and expectations are represented by weighted samples 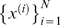


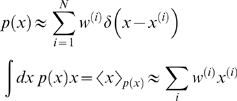
with 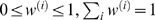. If samples are drawn from the distribution 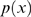 directly, the weights 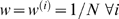. In the present case, this would mean drawing samples from the distributions 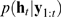 and 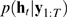, which is not possible because they themselves depend on integrals at adjacent timesteps which are hard to evaluate exactly. Instead, importance sampling allows sampling from a different “proposal” distribution 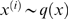 and compensating by setting 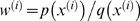. Here, we first seek samples and forward filtering weights 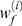 such that
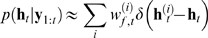
(15)and based on these will then derive backwards, smoothing weights such that
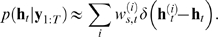
(16)Substituting the desideratum in equation 15 for time 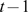 into equation 12
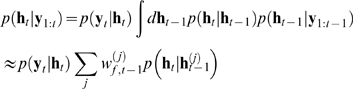
(17)As a proposal distribution for our setting we use the one-step predictive probability distribution (derived from the Markov property in equation 13):
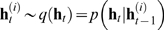
(18)where 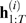 is termed the 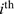 “particle”. The samples are made to reflect the conditional distribution by adjusting the weights, for which the probabilities 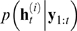 need to be computed. These are given by
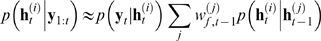
which involves a sum over 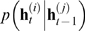 that is quadratic in 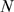. We approximate this by
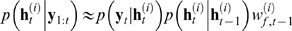
(19)which neglects the probability that the particle 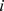 at time 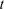 could in fact have arisen from particle 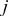 at time 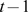. The weights for each of the particles are then given by a simple update equation:
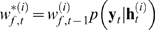
(20)

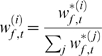
(21)


One well-known consequence of the approximation in equations 19–21 is that over time, the variance of the weights becomes large; this means that most particles have negligible weight, and only one particle is used to represent a whole distribution. Classically, this problem is prevented by resampling, and we here use stratified resampling [8]. This procedure, illustrated in Figure 2, results in eliminating particles that assign little, and duplicating particles that assign large likelihood to the data whenever the effective number of particles 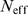 drops below some threshold, here 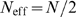.

It should be pointed out that it is also possible to interpolate between observations, or to do learning (see below) from subsampled traces. For example, assume we have a recording frequency of 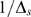 but wish to infer the underlying signal at a higher frequency 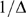, with 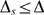. At time points without observation the likelihood term in equation 21 is uninformative (flat) and we therefore set
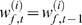
(22)keeping equation 21 for the remainder of times. In this paper, we will run compartmental models (equation 1) at sampling intervals 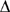, and recover signals to that same temporal precision from data subsampled at intervals 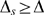. See e.g. [32] for further details on incorporating intermittently-sampled observations into the alternative predictive distribution 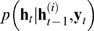.

We have so far derived the filtering weights such that particles are representative of the distribution conditioned on the past data 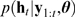. It often is more appropriate to condition on the entire set of measurements, i.e. represent the distribution 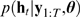. We will see that this is also necessary for the parameter inference in the M-step. Substituting equations 15 and 16 into equation 14, we arrive at the updates for the smoothing weights
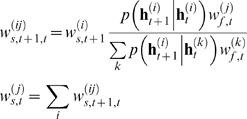
where the weights 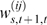 now represent the joint distribution of the hidden variables at adjacent timesteps:
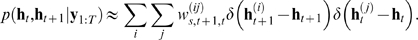



### Parameter inference

The maximum likelihood estimate of the parameters can be inferred via a maximisation of an expectation over the hidden variables:
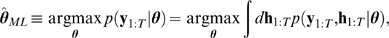
where 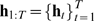. This is achieved by iterating over the two steps of the EM algorithm. In the M-step of the 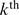 iteration, the likelihood of the entire set of measurements 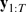 with respect to the parameters 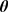 is maximised by maximising the expected total log likelihood [25]

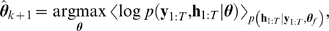
which is achieved by setting the gradients with respect to 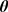 to zero (see [31],[33] for alternative approaches). For the main linear parameters we seek to infer in the compartmental model (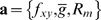), these equations are solved by performing a constrained linear regression, akin to that in equation 7. We write the total likelihood in terms of the dynamic and the observation models (equations 10 and 11):
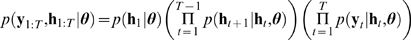
Let us assume that we have noisy measurements of the voltage. Because the parametrisation of the evolution of the voltage is linear, but that of the other hidden variables is not, we separate the two as 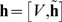 where 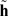 are the gates of the conductances affecting the voltage (a similar formulation can be written for [Ca^2+^] observations). Approximating the expectations by the weighted sums of the particles defined in the previous section, we arrive at
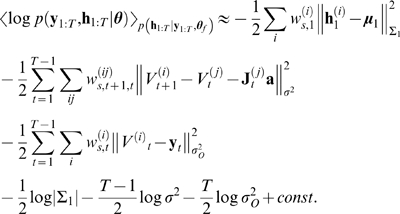
(23)where 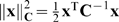, 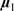 and 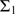 parametrise the distribution 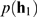 over the initial hidden variables at time 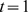, and 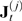 is the 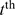 row of the matrix 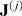 derived from particle 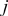. Note that because we are not inferring the kinetics of the channels, the evolution term for the gates (a sum over terms of the form 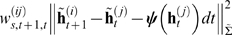) is a constant and can be neglected. Now setting the gradients of equation 23 with respect to the parameters to zero, we find that the linear parameters can be written, as in equation 7, as a straightforward quadratic minimisation with linear constraints
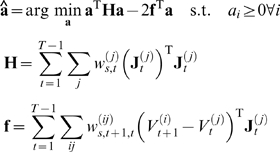
(24)where we see that the Hessian 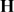 and the linear term 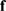 of the problem are given by an expectation involving the particles. Importantly, this is still a quadratic optimisation problem with linear constraints, and which is efficiently solved by standard packages. Similarly, the initialisation parameters for the unobserved hidden variables are given by
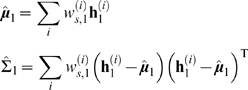
which are just the conditional mean and variance of the particles at time 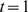; and the evolution and observation noise terms finally by
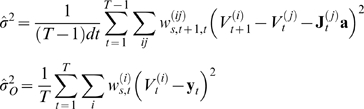
Thus, the procedure iterates over running the particle smoother in section *Sequential Monte Carlo* and then inferring the optimal parameters from the smoothed estimates of the unobserved variables.

## Results

### Model-based smoothing

We first present results on model-based smoothing. Here, we assume that we have a *correct* description of the parameters of the cell under scrutiny, and use this description to infer the true underlying signal from noisy measurements. These results may be considered as one possible application of a detailed model. Figure 2A shows the data, which was generated from a known, single-compartment cell with Hodgkin-Huxley-like conductances by adding Gaussian noise. The variance of the noise was chosen to replicate typical signal-to-noise ratios from voltage-dye experiments [2]. Figure 2B shows the 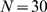 particles used here, and Figure 2C the number of particles with non-negligible weights (the “effective” number 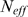 of particles). We see that when 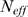 hits a threshold of 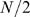, resampling results in large jumps in some particles. At around 3 ms, we see that some particles, which produced a spike at a time when there is little evidence for it in the data, are re-set to a value that is in better accord with the data. Figure 2D shows the close match between the true underlying signal and the inferred mean 
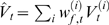
, while Figure 2E shows that even the unobserved channel open fractions are inferred very accurately. The match for both the voltage and the open channel fractions improves with the number of particles. Code for the implementation of this smoothing step is available online at http://www.gatsby.ucl.ac.uk/˜qhuys/code.html.

**Figure 3:**
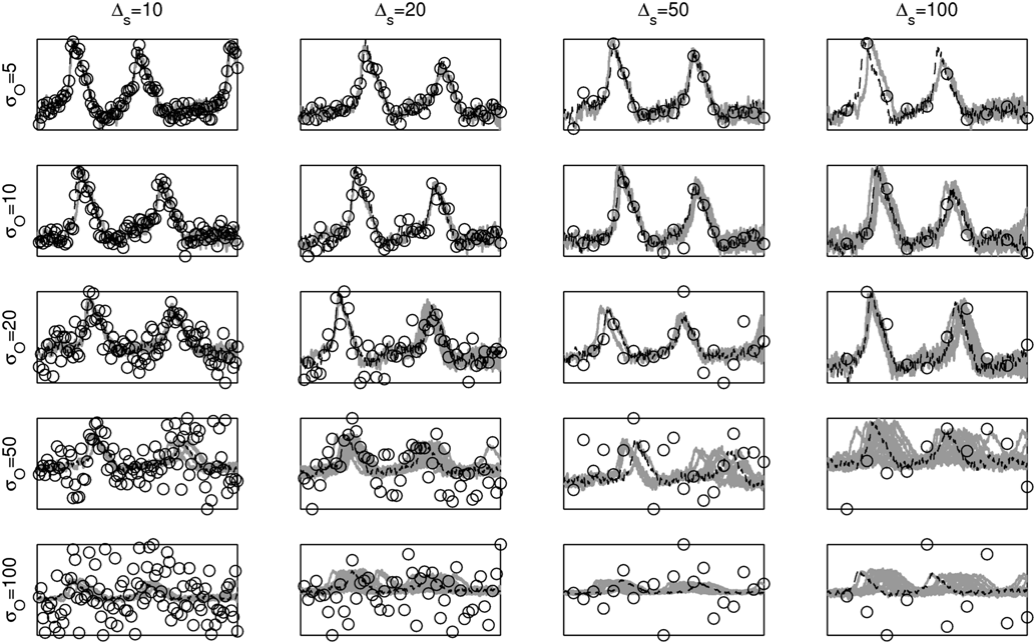
Performance of the model-based smoother with varying observation noise 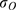 and temporal subsampling 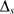. True underlying voltage trace in dashed black lines, the 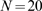 particles in gray and the data in black circles. Accurate inference of underlying voltage signals, and thus of spike times, is possible with accurate descriptions of the cell, over a wide range of noise levels and even at low sampling frequencies.

For imaging data, the laser often has to be moved between recording locations, leading to intermittent sampling at any one location (see [34]–[36]). Figure 3 illustrates the performance of the model-based smoother both for varying noise levels and for temporal subsampling. We see that even for very noisy and highly subsampled data, the spikes can be recovered very well.

**Figure 4:**
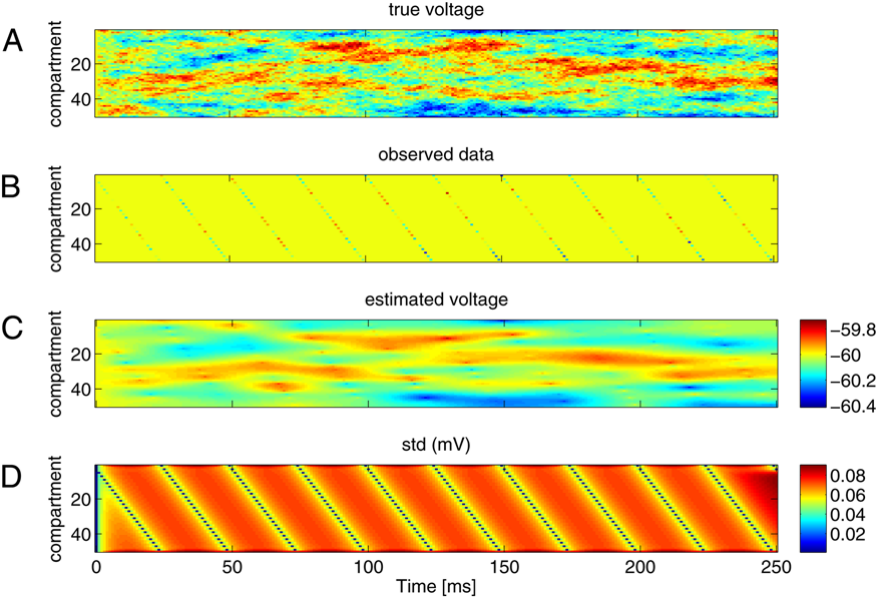
Inferring spatiotemporal voltage distribution from scanning, intermittent samples. A: True underlying voltage signal as a function of time for all 15 compartments. This was generated by injecting white noise current into a passive cell containing 50 linearly arranged compartments. B: Samples obtained by scanning repeatedly along the dendrite. The samples are seen as diagonal lines extending downwards, ie each compartment was sampled in sequence, overall 10 times and 25 ms apart. Note that the samples were noisy (*σ_O_* = 3.16 mV). C: Conditional expected voltage time course for all compartments reconstructed by Kalman smoothing. The colorbar indicates the voltage for all three panels. Note that even though there is only sparse data over time and space, a smooth version of the full spatiotemporal pattern is recovered. D: Variance of estimated voltage. It is smallest at the observation times and rapidly reaches a steady state between observations. Due to the smoothing, which takes future data into account, the variance diminishes *ahead* of observations.

Figure 4 shows a different aspect of the same issue, whereby the laser moves linearly across an extended linear dendrite. Here, samples are taken every 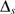 timesteps, but samples from each individual compartment are only obtained each 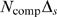. The true voltage across the entire passive dendrite is shown in Figure 4A, and the sparse data points distributed over the dendrite are shown in panel B. The inferred mean in panel C matches the true voltage very well. For this *passive*, linear example, the equations for the hidden dynamical system are exactly those of a Kalman smoother model [37]; thus the standard Kalman smoother performs the correct spatial and temporal smoothing once the parameters are known, with no need for the more general (but more computationally costly) particle smoother introduced above. More precisely, in this case the integrals in equations 12 and 14 can be evaluated analytically, and no sampling is necessary. The supplemental video S1 shows the results of a similar linear (passive-membrane) simulation, performed on a branched simulated dendrite (instead of the linear dendritic segment illustrated in Figure 4).

We emphasize that the strong performance of the particle smoother and the Kalman smoother here should not be surprising, since the data were generated from a known model and in these cases these methods perform smoothing in a statistically optimal manner. Rather, these results should illustrate the power of using an exact, correct description of the cell and its dynamics.

### EM – inferring cellular parameters

We have so far shown model-based filtering assuming that a full model of the cell under scrutiny is available. Here, we instead infer some of the main parameters from the data; specifically the linear parameters 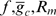, the observation noise 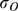 and the evolution noise 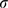. We continue to assume, however, that the kinetics of all channels that may be present in the cell are known exactly (see [23] for a discussion of this assumption).

**Figure 5:**
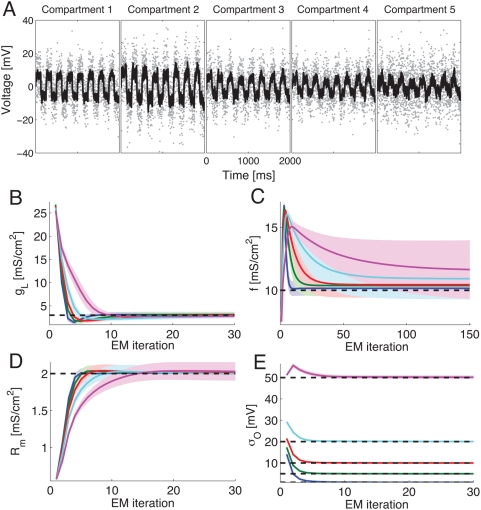
Inferring biophysical parameters from noisy measurements in a passive cell. A: True voltage (black) and noisy data (grey dots) from the 5 compartments of the cell with noise level *σ_O_* = 10 mV. B–E: Parameter inference with EM. Each panel shows the average inference time course±one st. dev. of one of the cellular parameters. B: Leak conductance; C: intercompartmental conductance; D: input resistivity; E: Observation noise variance. The grey dotted line shows the true values. The coloured lines show the inference for varying levels of noise 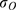. Blue: *σ_O_* = 1 mV, Green: *σ_O_* = 5 mV, Red: *σ_O_* = 10 mV, Cyan: *σ_O_* = 20 mV, Magenta: *σ_O_* = 50 mV. Throughout Δ*_s_* = 1 ms = 10Δ. Note that accurate estimation of the leak, input resistance and noise levels is even possible when the noise is five times as large as that shown in panel A. Inference of the intercompartmental conductance suffers most from the added noise because the small intercompartmental currents have to be distinguished from the apparent currents arising from noise fluctuations in the observations from neighbouring compartments. Throughout, the underlying voltage was estimated highly accurately (data not shown), which is also reflected in the accurate estimates of 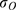.

Figure 5 illustrates the inference for a passive multicompartmental model, similar to that in Figure 4, but driven by a square current injection into the second compartment. Figure 5B shows statistics of the inference of the leak conductance maximal density 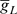, the intercompartmental conductance 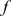, the input resistance 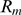 and the observation noise 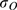 across 50 different randomly generated noisy voltage traces. All the parameters are reliably recovered from 2 seconds of data at a 1 ms sampling frequency.

**Figure 6:**
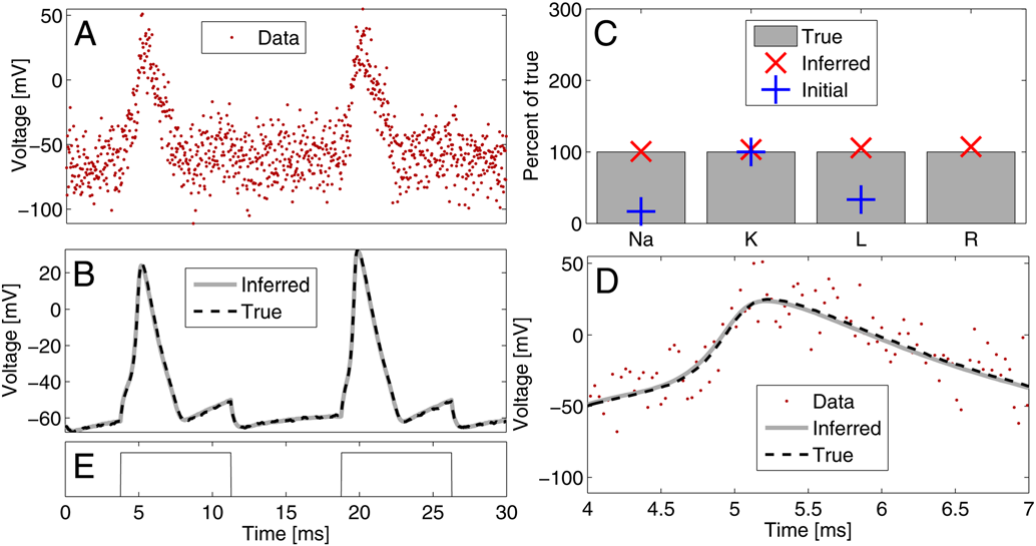
Example inference for single compartment with active conductances. A: Noisy data, *σ_O_* = 10 mV; B: True underlying voltage (black dashed line) resulting from current pulse injection shown in E. The gray trace shows the mean inferred voltage after inferring the paramter values in C. C: Initial (blue +) and inferred parameter values (red ×) in percent relative to true values (gray bars *ḡ* *_Na_* = 120 mS/cm^2^, *ḡ* *_K_* = 20 mS/cm^2^, *ḡ* *_Leak_* = 3 mS/cm^2^, *R_m_* = 1 mS/cm^2^). At the initial values the cell was non-spiking. D: Magnified view showing data, inferred and true voltage traces for the first spike. Thus, despite the very high noise levels and an initially inaccurate, non-spiking model of the cell, knowledge of the channel kinetics allows accurate inference of the channel densities and very precise reconstruction of the underlying voltage trace.

**Figure 7:**
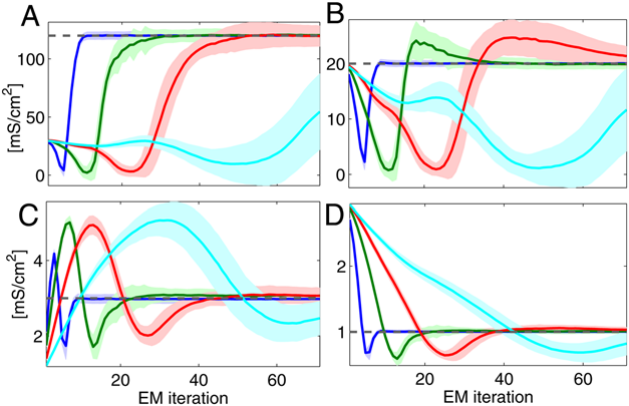
Time course of parameter estimation with HH channels. The four panels show, respectively, the inference for the conductance parameters A: 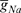 B: 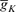 C: 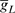 and D: 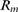. The thick coloured lines indicate the mean over 50 data samples and the shaded areas 1 st. dev. The colours indicate varying noise levels 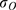. Blue: *σ_O_* = 1 mV, Green: *σ_O_* = 5 mV, Red: *σ_O_* = 10 mV, Cyan: *σ_O_* = 20 mV. The true parameters are indicated by the horizontal gray dashed lines. Throughout Δ*_s_* = Δ = 0.02 ms. The main effect of increasing observation noise is to slow down the inference. In addition, larger observation noise also adds variance to the parameter estimates. Throughout, only 10 ms of data were used.

We now proceed to infer channel densities and observation noise from active compartments with either four or eight channels. Figure 6 shows an example trace and inference for the four channel case (using Hodgkin-Huxley like channel kinetics). Again, we stimulated with square current pulses. Only 10 ms of data were recorded, but at a very high temporal resolution Δ*_s_* = Δ = 0.02 ms. We see that both the underlying voltage trace and the channel and input resistance are recovered with high accuracy. Figure 7 presents batch data over 50 runs for varying levels of observation noise 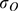. The observation noise here has two effects: first, it slows down the inference (as every data point is less informative), but secondly the variance across runs increases with increasing noise (although the mean is still accurate). For illustration purposes, we started the maximal K^+^ conductance at its correct value. As can be seen, however, the inference initially moves 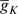 *away* from the optimum, to compensate for the other conductance misestimations. (This nonmonotonic behavior in 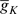 is a result of the fact that the EM algorithm is searching for an optimal setting of all of the cell's conductance parameters, not just a single parameter; we will return to this issue below.)

**Figure 8:**
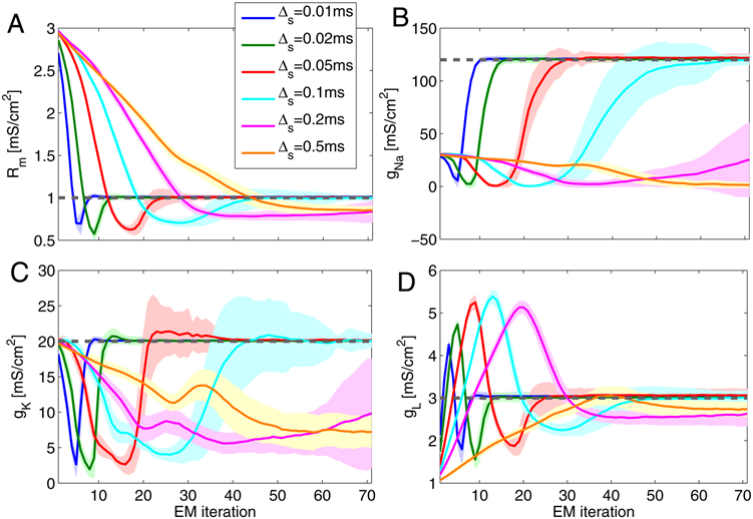
Subsampling slows down parametric inference. Inference of the same parameters as in previous Figure (A: 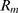, B: 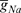, C: 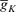, D: 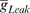), but the different colours now indicate increasing subsampling. Particles evolved at timesteps of Δ = 0.04 ms. The coloured traces inference with show sampling timesteps of Δ*_s_* = {0.01,0.02,0.05,0.1,0.5} ms respectively. All particles were run with a Δ = 0.01 ms timestep, and the total recording was always 10 ms long, meaning that progressive subsampling decreased the total number of data points. Thus, it can be seen that parameter inference is quite relatively to undersampling. At very large subsampling times, 10 ms of data supplied too few observations during a spike to justify inference of high levels of Na^+^ and K^+^ conductances, but the input resistance and the leak were still reliably and accurately inferred.

Parametric inference here has so far employed densely sampled traces (see Figure 6A). The algorithm however applies equally to subsampled traces (see equation 22). Figure 8 shows the effect of subsampling. We see that subsampling, just as noise, slows down the inference, until the active conductances are no longer inferred accurately (the yellow trace for Δ*_s_* = 0.5 ms). In this case, the total recording length of 10 ms meant that inference had to be done based on one single spike. For longer recordings, information about multiple spikes can of course be combined, partially alleviating this problem; however, we have found that in highly active membranes, sampling frequencies below about 1 KHz led to inaccurate estimates of sodium channel densities (since at slower sampling rates we will typically miss significant portions of the upswing of the action potential, leading the EM algorithm to underestimate the sodium channel density). Note that we kept the length of the recording in Figure 8 constant, and thus subsampling reduced the total number of measurements.

**Figure 9:**
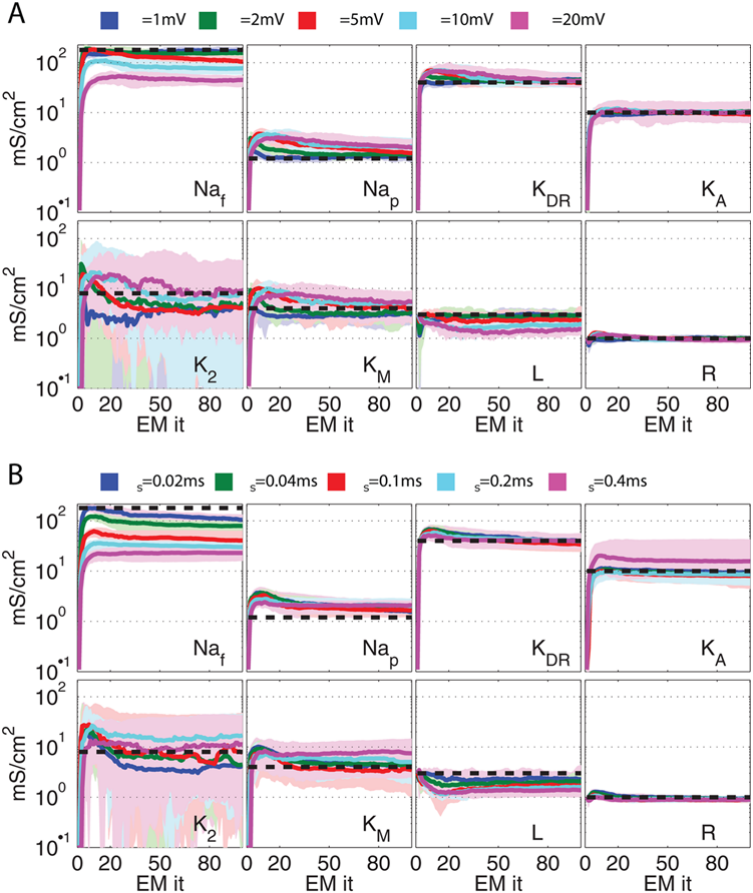
Time course of parameter estimation in a model with eight conductances. Evolution of estimates of channel densities for compartment with eight channels. Colours show inference with changes in the observation noise 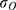 and the subsampling 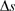. True levels are indicated by dotted gray lines. A: Δ*_s_* = .02 ms, *σ_O_* = {1,2,5,10,20} mV respectively for blue, green, red, cyan and purple lines B: *σ_O_* = 5 mV, Δ*_s_* = {.02,.04,.1,.2,.4} ms again for blue, green, red, cyan and purple lines respectively. Thick lines show median, thin lines show 10 and 90% quantiles of distribution across 50 runs.

As with any importance sampling method, particle filtering is known to suffer in higher dimensions [38]. To investigate the dependence of the particle smoother's accuracy on the dimensionality of the state space, we applied the method to a compartment with a larger number of channels: fast (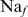) and persistent Na^+^ (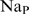) channels in addition to leak (L) and delayed rectivier (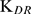), A-type (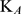), K2-type (K_2_) and M-type (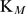) K^+^ channels (channel kinetics from ModelDB [39], from [9],[40]). Figure 9 shows the evolution of the channel intensities during inference. Estimates of most channel densities are correct up to a factor of approximately 2. Unlike in the previous, smaller example, as either observation noise or subsampling increase, significant biases in the estimation of channel densities appear. For instance, the density of the fast sodium channel observed with noise of standard deviation 20 mV is only about half the true value.

It is worth noting that this bias problem is not observed in the passive linear case, where the analytic Kalman smoother suffices to perform the inference: we can infer the linear dynamical parameters of neurons with many compartments, as long as we sample information from each compartment [23]. Instead, the difficulty here is due to multicollinearity of the regression performed in the M-step of the EM algorithm and to the fact that the particle smoother leads to biased estimation of covariance parameters in high-dimensional cases [38]. We will discuss some possible remedies for these biases below.

**Figure 10:**
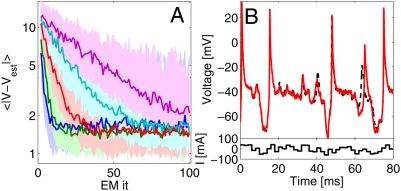
Predictive performance of inferred parameter settings on new input current. A: Parameter estimates as shown in Figure 9A were used to predict response to a new input stimulus. The plot shows the absolute error averaged over the entire trace (3000 timesteps, Δ*_t_* = .02 ms), for 40 runs. Thick lines show the median, shaded areas 10 and 90% quantiles over the same 40 runs as in Figure 9. Blue: *σ_O_* = 1 mV, Green: *σ_O_* = 2 mV, Red: *σ_O_* = 5 mV, Cyan: *σ_O_* = 10 mV, Magenta: *σ_O_* = 20 mV. Note logarithmic y axis. B: Example prediction trace. The dashed black line shows the response of the cell with the true parameters, the red line that with the inferred parameters. The observation noise was *σ_O_* = 20 mV, while the average error for this trace 〈|*V*−*V_est_*|〉 = 2.96 mV.

Somewhat surprisingly, however, these observed estimation biases do not prove catastrophic if we care about predicting or smoothing the subthreshold voltage. Figure 10A compares the response to a new, random, input current of a compartment with the true parameters to that of a compartment with parameters as estimated during EM inference, while Figure 10B shows an example prediction with 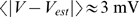. Note the large plateau potentials after the spikes due to the persistent sodium channel 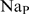. We see that even the parameters as estimated under high noise accurately come to predict the response to a new, previously unseen, input current. The asymptote in Figure 10A is determined by the true evolution noise level (here *σ* = 1 mV): the more inherent noise, the less a response to a specific input is actually predictable.

**Figure 11:**
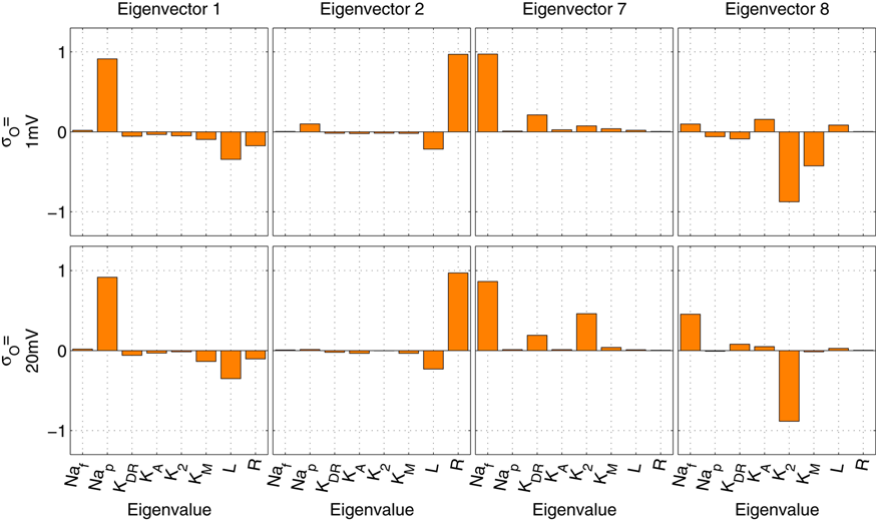
Eigenstructure of Hessian 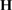 with varying observation noise. Eigenvector 1 has the largest (>10^4^), and eigenvector 8 respectively the smallest eigenvalue (∼0.5). Independently of the noise, the smalles eigenvectors involve those channels for which inference in Figure 9 appeared least reliable: the fast Na^+^ and the K_2_-type K^+^ channel.

Some further insight into the problem can be gained by looking at the structure of the Hessian of the total likelihood 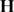 around the true parameters. We estimate 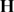 by running the particle smoother with a large number of particles once at the true parameter value; more generally, one could perform a similar analysis about the inferred parameter setting to obtain a parametric bootstrap estimate of the posterior uncertainty. Figure 11 shows that, around the true value, changes in either the fast Na^+^ or the K_2_-type K^+^ channel have the least effect; i.e., the curvature in the loglikelihood is smallest in these directions, indicating that the observed data does not adequately constrain our parameter estimates in these directions, and prior information must be used to constrain these estimates instead. This explains why these channels showed disproportionately large amounts of inference variability, and why the prediction error did not suffer catastrophically from their relatively inaccurate inference (Figure 10A). See [23] for further discussion of this multicollinearity issue in large multichannel models.

### Estimation of subthreshold nonlinearity by nonparametric EM

We saw in the last section that as the dimensionality of the state vector 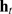 grows, we may lose the ability to simultaneously estimate all of the system parameters. How can we deal with this issue? One approach is to take a step back: in many statistical settings we do not care primarily about estimating the underlying model parameters accurately, but rather we just need a model that predicts the data well. It is worth emphasizing that the methods we have intrduced here can be quite useful in this setting as well. As an important example, consider the problem of estimating the subthreshold voltage given noisy observations. In many applications, we are more interested in a method which will reliably extract the subthreshold voltage than in the parameters underlying the method. For example, if a linear smoother (e.g., the Kalman smoother discussed above) works well, it might be more efficient and stable to stick with this simpler method, rather than attempting to estimate the parameters defining the cell's full complement of active membrane channels (indeed, depending on the signal-to-noise ratio and the collinearity structure of the problem, the latter goal may not be tractable, even in cases where the voltage may be reliably measured [23]).

Of course, in many cases linear smoothers are not appropriate. For example, the linear (Kalman) model typically leads to oversmoothing if the voltage dynamics are sufficiently nonlinear (data not shown), because action potentials take place on a much faster timescale than the passive membrane time constant. Thus it is worth looking for a method which can incorporate a flexible nonlinearity and whose parameters may not be directly interpretable biophysically but which nonetheless leads to good estimation of the signal of interest. We could just throw a lot of channels into the mix, but this increases the dimensionality of the state space, hurting the performance of the particle smoother and leading to multicollinearity problems in the M-step, as illustrated in the last subsection.

A more promising approach is to fit nonlinear dynamics directly, while keeping the dimensionality of the state space as small as possible. This has been a major theme in computational neuroscience, where the reduction of complicated multichannel models into low-dimensional models, useful for phase plane analysis, has led to great insights into qualitative neural dynamics [26],[41].

As a concrete example, we generated data from a strongly nonlinear (Fitzhugh-Nagumo) two-dimensional model, and then attempted to perform optimal smoothing, without prior knowledge of the underlying voltage nonlinearity. We initialized our analysis with a linear model, and then fit the nonlinearity nonparametrically via a straightforward nonparametric modification of the EM approach developed above.

**Figure 12:**
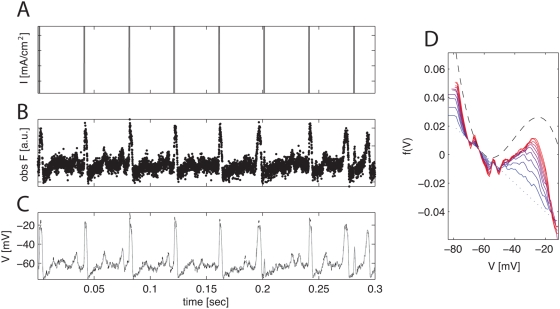
Estimating subthreshold nonlinearity via nonparametric EM, given noisy voltage measurements. A, B: input current and observed noisy voltage fluorescence data. C: inferred and true voltage trace. Black dashed trace: true voltage; gray solid trace: voltage inferred using nonlinearity given tenth EM iteration (red trace from right panel). Note that voltage is inferred quite accurately, despite the significant observation noise. D: voltage nonlinearity estimated over ten iterations of nonparametric EM. Black dashed trace: true nonlinearity; blue dotted trace: original estimate (linear initialization); solid traces: estimated nonlinearity. Color indicates iteration number: blue trace is first and red trace is last.

**Figure 13:**
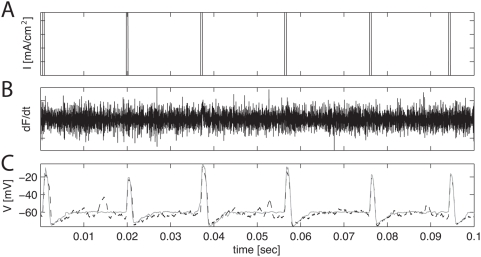
Estimating voltage given noisy calcium measurements, with nonlinearity estimated via nonparametric EM. A: Input current. B: Observed time derivative of calcium-sensitive fluorescence. Note the low SNR. C: True and inferred voltage. Black dashed trace: true voltage; gray solid trace: voltage inferred using nonlinearity following five EM iterations. Here the voltage-dependent calcium current had an activation potential at −20 mV (i.e., the calcium current is effectively zero at voltages significantly below −20 mV; at voltages >10 mV the current is ohmic). The superthreshold voltage behavior is captured fairly well, as are the post-spike hyperpolarized dynamics, but the details of the resting subthreshold behavior are lost.

In more detail, we generated data from the following model [41]:
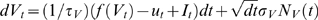
(25)

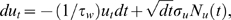
(26)where the nonlinear function 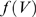 is cubic in this case, and 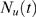 and 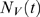 denote independent white Gaussian noise processes. Then, given noisy observations of the voltage 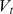 (Figure 12, left middle panel), we used a nonparametric version of our EM algorithm to estimate 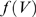. The E-step of the EM algorithm is unchanged in this context: we compute 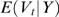 and 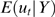, along with the other pairwise sufficient statistics, using our standard particle forward-backward smoother, given our current estimate of 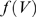. The M-step here is performed using a penalized spline method [42]: we represent 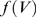 as a linearly weighted combination of fixed basis functions 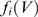:
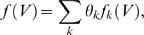
and then determine the optimal weights 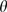 by maximum penalized likelihood:
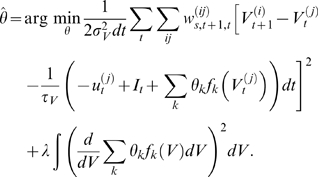
The first term here corresponds to the expected complete loglikelihood (as in equation (23)), while the second term is a penalty which serves to smooth the inferred function 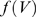 (by penalizing non-smooth solutions, i.e., functions 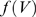 whose derivative has a large squared norm); the scalar 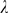 serves to set the balance between the smoothness of 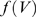 and the fit to the data. Despite its apparent complexity, in fact this expression is just a quadratic function of 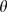 (just like equation (24)), and the update 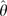 may be obtained by solving a simple linear equation. If the basis functions 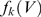 have limited overlap, then the Hessian of this objective function with respect to 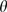 is banded (with bandwidth equal to the degree of overlap in the basis functions 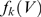), and therefore this linear equation can be solved quickly using sparse banded matrix solvers [42],[43]. We used 50 nonoverlapping simple step functions to represent 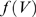 in Figures. 12–13, and each M-step took negligible time (≪1 sec). The penalty term 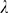 was fit crudely by eye here (we chose a 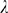 that led to a reasonable fit to the data, without drastically oversmoothing 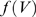); this could be done more systematically by model selection criteria such as maximum marginal likelihood or cross-validation, but the results were relatively insensitive to the precise choice of 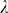. Finally, it is worth noting that the EM algorithm for maximum penalized likelihood estimation is guaranteed to (locally) optimize the penalized likelihood, just as the standard EM algorithm (locally) optimizes the unpenalized likelihood.

Results are shown in Figures 12 and 13. In Figure 12, we observe a noisy version of the voltage 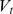, iterate the nonparametric penalized EM algorithm ten times to estimate 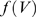, then compute the inferred voltage 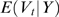. In Figure 13, instead of observing the noise-contaminated voltage directly, we observe the internal calcium concentration. This calcium concentration variable 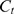 followed its own noisy dynamics,
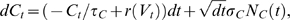
where 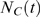 denotes white Gaussian noise, and the 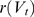 term represents a fast voltage-activated inward calcium current which activates at −20 mV (i.e., this current is negligible at rest; it is effectively only activated during spiking). We then observed a noisy fluorescence signal 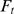 which was linearly related to the calcium concentration 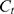 [32]. Since the informative signal in 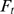 is not its absolute magnitude but rather how quickly it is currently changing (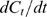 is dominated by 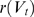 during an action potential), we plot the time derivative 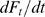 in Figure 13; note that the effective signal-to-noise in both Figures 12 and 13 is quite low.

The nonparametric EM-smoothing method effectively estimates the subthreshold voltage 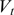 in each case, despite the low observation SNR. In Figure 12, our estimate of 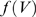 is biased towards a constant by our smoothing prior; this low-SNR data is not informative enough to overcome the effect of the smoothing penalty term here; indeed, since this oversmoothed estimate of 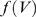 is sufficient to explain the data well, as seen in the left panels of Figure 12, the smoother estimate is preferred by the optimizer. With more data, or a higher SNR, the estimated 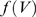 becomes more accurate (data not shown). It is also worth noting that if we attempt to estimate 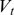 from 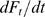 using a linear smoother in Figure 13, we completely miss the hyperpolarization following each action potential; this further illustrates the advantages of the model-based approach in the context of these highly nonlinear dynamical observations.

## Discussion

This paper applied standard machine learning techniques to the problem of inferring biophysically detailed models of single neurones automatically and directly from single-trial imaging data. In the first part, the paper presented techniques for the use of detailed models to filter noisy and temporally and spatially subsampled data in a principled way. The second part of the paper used this approach to infer unknown parameters by EM.

Our approach is somewhat different from standard approaches in the cellular computational neuroscience literature ([12],[14],[15],[19], although see [18]), in that we argue that the inference problem posed is equivalent to problems in many other statistical situations. We thus postulate a full probabilistic model of the observations and then use standard machine learning tools to do inference about biophysically relevant parameters. This is an approach that is more standard in other, closely related fields in neuroscience [44],[45]. Importantly, we attempt to use the description of the problem in detail to arrive at as efficient as possible a method of using the data. This implies that we directly compare recording traces (the voltage or calcium trace), rather than attempting to fit measures of the traces such as the ISI distribution, and the sufficient statistics that are used for the parameter inference involves aspects of the data these parameters influence directly. One alternative is to include a combination of such physiologically relevant objective functions and to apply more general fitting routines [46],[47]. A key assumption in our approach is that accurately fitting the voltage trace will lead to accurate fits of such other measures derived from the voltage trace, such as the inter-spike interval distribution. In the present approach this means that variability is explicitly captured by parameters internal to the model. In our experience, this is important to avoid both overfitting individual traces and neglecting the inherently stochastic nature of neural responses.

A number of possible alternatives to sequential Monte Carlo methods exist, such as variations of Kalman filtering like extended or unscented Kalman filters [48],[49], variational approaches (see [50]) and approximate innovation methods [45],[51],[52]. We here opted for a sequential Monte Carlo method because it has the advantage of allowing the approximation of arbitrary distributions and expectations. This is of particular importance in the problem at hand because a) we specifically wish to capture the nonlinearities in the problem as well as possible and b) the distributions over the unobserved states are highly non-Gaussian, due to both the nonlinearities but also due to unit bounds on the gates.

Model-based smoothing thus provides a well-founded alternative to standard smoothing techniques, and, importantly, allows smoothing of data without any averaging over either multiple cells or multiple trials [53]. This allows the inference of unobserved variables that have an effect on the observed variable. For example, just as one can infer the channels' open fractions, one can estimate the voltage from pure [Ca^2+^] recordings (data not shown). The formulation presented makes it also straightforward to combine measurements from various variables, say [Ca^2+^] and transmembrane voltage, simply by appropriately defining the observation density 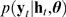. We should emphasize, though, that the techniques themselves are not novel. Rather, this paper aims to point out to what extent these techniques are promising for cellular imaging.

The demand, when smoothing, for an accurate knowledge of the cell's parameters is addressed in the learning part of the paper where some of the important parameters are inferred accurately from small amounts of data. One instructive finding is that adding noise to the observations did not hurt our inference on average, though it did make it slower and more variable (note the wider error bars in Figure 7). In the higher-dimensional cases, we found that the dimensions in parameter space which have least effect on the models' behavior were also least well inferred. This may replicate the reports of significant flat (although not disconnected) regions in parameter space revealed in extensive parametric fits using other methods [19]. A number of parameters also remain beyond the reach of the methods discussed here, notably the kinetic channel parameters; this is the objective of the non-parametric inference in the last section of the *Results*, and also of further ongoing work.

A number of additional questions remain open. Perhaps the fundamental direction for future research involves the analysis of models in which the nonlinear hidden variable 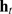 is high-dimensional. As we saw in section *EM – inferrring cellular parameters*, our basic particle smoothing-EM methodology can break down in this high-dimensional setting. The statistical literature suggests two standard options here. First, we could replace the particle smoothing method with more general (but more computationally expensive) Markov chain Monte Carlo (MCMC) methods [54] for computing the necessary sufficient statistics for inference in our model. Designing efficient MCMC techniques suitable for high-dimensional multicompartmental neural models remains a completely open research topic. Second, to combat the multicollinearity diagnosed in Figure 11 (see also Figure 6 of [23]), we could replace the maximum-likelihood estimates considered here with maximum a posteriori (maximum penalized likelihood) estimates, by incorporating terms in our objective function (7) to penalize parameter settings which are believed to be unlikely a priori. As discussed in section *Estimation of subthreshold nonlinearity by nonparametric EM*, the EM algorithm for maximum penalized likelihood estimation follows exactly the same structure as the standard EM algorithm for maximum likelihood estimation, and therefore our methodology may easily be adapted for this case. Finally, a third option is to proceed along the direction indicated in section *Estimation of subthreshold nonlinearity by nonparametric EM*: instead of attempting to fit the parameters of our model perfectly, in many cases we can develop good voltage smoothers using a cruder, approximate model whose parameters may be estimated much more tractably. We expect that a combination of these three strategies will prove to be crucial as optimal filtering of nonlinear voltage- and calcium-sensitive dendritic imaging data becomes more prevalent as a basic tool in systems neuroscience.

## Supporting Information

Video S1 Kalman smoother video. The video shows the inference of the underlying voltage in a passive cell from intermittent recordings along the dendrites. The left panel shows the true voltage; the middle panel the measurements (black means no measurement from that dendritic location at that time, cf. Figure 4); and the right panel the reconstructed voltage in the entire cell.
(1.63 MB MOV)
